# Biostrap Kairos Wristband Versus Electrocardiography for Resting Heart Rate Variability Assessment

**DOI:** 10.3390/s25103165

**Published:** 2025-05-17

**Authors:** Andrew A. Flatt, Ann Claire E. Blalock, Allison N. Wade, Bryan L. Riemann

**Affiliations:** Biodynamics and Human Performance Center, Department of Health Sciences and Kinesiology, Georgia Southern University, Armstrong Campus, 11935 Abercorn St., Savannah, GA 31419, USA

**Keywords:** wearable technology, m-health, cardiovascular, device validation, autonomic nervous system

## Abstract

The Kairos wristband offers on-demand heart rate variability (HRV) assessment through its “Spot Check” feature, enabling standardized recordings for clinical, research, or self-tracking purposes, but its validity is untested. Therefore, we compared the Kairos wristband to electrocardiography (ECG) for resting HRV assessment in young adults, and investigated the influence of skin pigmentation (M-index) on measurement accuracy. Simultaneous 3 min Kairos and ECG samples were obtained in the supine (n = 32) and seated (n = 30) position. Comparisons included resting heart rate (RHR) and time domain (root-mean square of successive differences [RMSSD], standard deviation of normal RR intervals [SDNN]), frequency domain (low [LF] and high frequency [HF]), and non-linear (standard deviation 1 [SD1] and SD2) HRV metrics. RHR showed excellent agreement whereas HF, LF, and SD2 showed poor agreement. For the remaining metrics, SDNN showed the strongest absolute and relative agreement, followed by SD1 and RMSSD. However, most HRV metrics exhibited heteroscedasticity or proportional bias, with greater error and underestimation at higher HRV values. M-index was unrelated to method difference scores, except for seated SD2 (*p* = 0.01). The Kairos wristband can be used to measure RHR, but HRV assessment should be limited to SDNN for global variability and SD1 or RMSSD for cardiac–parasympathetic activity. However, these metrics should be interpreted within the level of agreement identified in this study, and with consideration of the observed trend of diminished accuracy with higher HRV values.

## 1. Introduction

The autonomic nervous system (ANS) influences cardiac function through sympathetic and parasympathetic innervation. As an effector organ involved in homeostatic regulation, cardiac activity is modulated by the ANS on a beat-by-beat basis, quantified as heart rate variability (HRV) [1]. Serial tracking of resting heart rate (RHR) and HRV reflects changes in cardiac ANS control, and has important health and performance implications. For example, serial self-tracking in cardiac patients using a valid chest-strap monitoring system revealed distinct day-to-day trends that preceded a cardiac event, in contrast to event-free patients, suggesting potential implications for early medical intervention [2]. In athletic contexts, serial self-tracking with a valid chest-strap monitoring system or finger photoplethysmography (PPG) device revealed adverse changes in ANS status preceding illness and overtraining, and following a concussion, emphasizing the potential for early detection, prevention efforts, and ongoing monitoring of status [3,4,5]. Finally, in the general adult population, HRV data collected from self-tracking devices has been linked to physical activity patterns and various cardiometabolic risk factors, suggesting its potential as a behavior modification tool for improving health outcomes [6,7].

Wearable devices, such as wristbands and smartwatches, have drastically increased the popularity of self-tracking by making HR monitoring more accessible. For example, one-third of American adults utilize wearable technology capable of tracking heart rate via PPG [8]. Moreover, American College of Sports Medicine (ACSM) survey findings have consistently reported wearable technology as the top health and fitness trend in recent years [9]. Despite increasingly wide-spread usage, third-party validation studies are limited, and may not exist yet for newer products. Previous research into the accuracy of commercial wristbands have produced mixed findings [10]. Furthermore, studies [11,12,13,14] that exported inter-pulse interval data from the wristband for external processing and HRV calculation do not test the accuracy of the wristbands smartphone application. This limits the findings given that most users will rely on values computed by the wearable device system. Additionally, many studies have neglected to examine whether accuracy is impacted by skin tone. Darker skin has been associated with greater PPG error in recent investigations due to differences in light absorption and photon scattering density and detection at varying wavelengths [15,16]. Thus, further validation studies are needed that assess the internal processing and computing of HRV by the wearable device system, and that address inter-individual differences in skin tone or pigmentation.

In 2023, a biosensor company named Biostrap introduced the Kairos wristband to the market. Unlike many other consumer wristband products, Kairos and the accompanying Vital Science smartphone application enable users to perform on-demand RHR and HRV measurements via their “Spot Check” feature. The Spot Check involves a 3-min sampling period under static, resting conditions, after which a report is automatically generated and emailed to the user. This report includes numerous time domain, frequency domain, and non-linear HRV metrics, significantly enhancing the functionality of the device. As a result, it allows for standardized HRV measurements that are applicable in various athletic, clinical, and research settings. To date, the accuracy of the Kairos “Spot Check” feature has not undergone third-party investigation. Therefore, we aimed to (1) compare the Kairos wristband with gold standard electrocardiography (ECG) for HRV assessment, and (2) investigate how skin pigmentation may impact the accuracy of the wristband’s HRV estimations.

## 2. Materials and Methods

### 2.1. Experimental Design

We used a cross-sectional study design to evaluate the accuracy of the Kairos wristband (Biostrap USA, LLC, Austin, TX, USA) for RHR and HRV assessment compared to gold-standard ECG. Data collection occurred in a single session within a controlled laboratory environment. All participants completed simultaneous 3 min recordings via Kairos and ECG in both supine and seated positions to assess RHR and HRV under different postural conditions. Each 3 min recording was preceded by at least 1 min for stabilization. Wrist skin pigmentation was subsequently quantified to assess its potential association with Kairos measurement error.

### 2.2. Participants

A racially diverse sample of apparently healthy young men and women (n = 40) were recruited for this study. To participate, individuals needed to be between the ages of 18 and 39 years and free from known cardiovascular, metabolic, and neurological disorders. In addition, individuals were required to have no tattoos or scarring of the skin at the site of wristband placement. Prior to data collection, prospective participants were informed of the risks and benefits of the study, and were given the opportunity to ask questions before signing a consent form. The experimental protocol was granted ethical approval by the Georgia Southern University Institutional Review Board (Protocol H23179).

### 2.3. Electrocardiography

All participants abstained from food and fluid ingestion for ≥2 h prior to testing [17]. Data collection procedures were carried out in a climate controlled laboratory (21 °C, 40% humidity) with ambient lighting. Height and mass were measured with a wall-mounted stadiometer and calibrated digital scale, respectively. Criterion HRV was derived from wireless ECG recordings (MP 160, Biopac Systems Inc., Coletta, CA, USA). Disposable Ag-AgCl electrodes were placed on the skin of the trunk in a modified lead II configuration. Sampling frequency was set at 1000 Hz. Real-time cardiac cycles were displayed on a laptop computer with AcqKnowledge 5.0 software (Biopac Systems Inc., Coletta, CA, USA), which was used to mark the ECG at start and finish times of simultaneous 3 min recordings with the wristband. Kubios HRV Scientific software (Version 4.1.0, University of Kuopio, Kuopio, Finland) was used to manually detect ECG abnormalities and compute HRV parameters. Since filtering and correction procedures can confound the agreement between devices, we only included ECG samples with a clear signal and 0 ectopic beats. Thus, included samples contained 100% normal beats from sinus origin, requiring no R-R interval filtering. Prior to HRV analysis, R-R intervals were detrended using the smoothness priors method [18] to maintain consistency with Kairos procedures. Time domain (resting heart rate [RHR], root-mean square of successive differences [RMSSD], standard deviation of normal R-R intervals [SDNN]), frequency domain (high frequency [HF], low frequency [LF] spectral power using a fast Fourier transformation), and non-linear (standard deviation of points perpendicular to the line of identity in the Poincaré plot [SD1], standard deviation of points along the line of identity in the Poincaré plot [SD2]) HRV parameters were computed and recorded for analysis. These are the same parameters computed by the Kairos system. In general, RMSSD, HF, and SD1 represent cardiac–parasympathetic modulation, SDNN reflects global variability with influence from parasympathetic and sympathetic modulation, and LF and SD2 reflect baroreflex activity [19]. Reduced HRV parameters are observed in or precede various clinical conditions, and can provide prognostic information. For example, lower time domain markers such as RMSSD and SDNN precede hypertension [20], time and frequency (HF, LF) domain parameters precede diabetes [21], and reduced parasympathetic parameters (RMSSD, HF) predict accelerated cognitive decline [22]. Additionally, among cardiac and cancer patients, those with higher HRV tend to show longer survival [23,24]. Although non-linear parameters (SD1, SD2) are less frequently utilized, their strong correlation with time and frequency domain parameters suggests they provide similar insights [25,26].

### 2.4. Wristband

**Figure 1:**
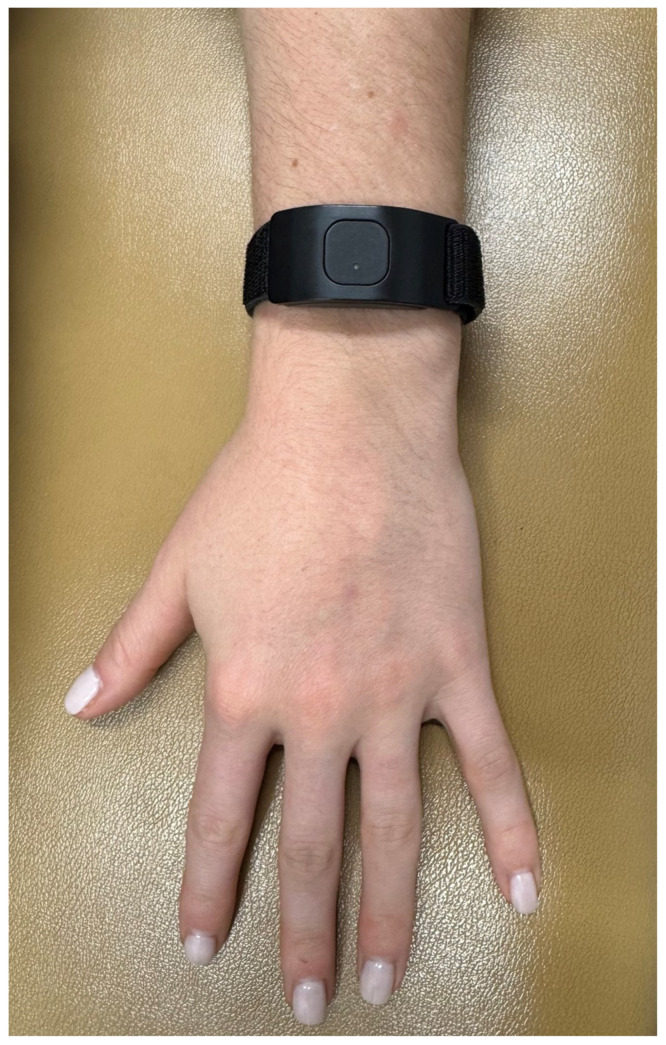
Photograph of a participant wearing the Biostrap Kairos wristband while resting in the supine position.

The Kairos wristband was fitted around the left wrist of the participant (Figure 1) according to manufacturer specifications. An iPhone 13 (Apple Inc. Cupertino, CA, USA) was paired with the wristband via Bluetooth. The wristband collects raw PPG data using a high-sensitivity complementary metal-oxide-semiconductor optical sensor with two green and one infrared light emitting diodes. The accompanying Vital Science mobile application (Version 1.1.26, Biostrap USA LLC, Austin, TX, USA) was used to perform 3-min recordings using the “Spot Check” feature. Supine recordings were performed on a comfortable examination table with the wrist in a pronated position. Seated recordings were performed in a back-supported chair with hands resting in a pronated position on their lap. Seated HRV measurements are common for at-home and remote assessments [27] but the upright position may affect the accuracy of PPG devices [28]. A stabilization period of at least 1 min preceded each recording [29]. Participants were instructed to remain quiet, still, and to breathe naturally during measurements. Once a Spot Check was completed, PPG data were automatically transmitted to cloud servers by the Vital Science application for processing and calculation of HRV parameters. Results were provided in a PDF report, which was automatically emailed to the registered user. Only samples with adequate signal quality, as indicated on the PDF report, were included in the analysis.

### 2.5. Skin Pigmentation

Following HRV data collection, the melanin index (M-index) was quantified using reflectance spectrophotometry (DermaSpectrometer; Cortex Technology, Hadsund, Denmark). Measurements were taken on the dorsal aspect of the wrist, corresponding to the PPG sampling site. This assessment location was chosen to evaluate the interaction between skin pigmentation and the accuracy of HRV estimation from the wristband. Higher M-index values indicate greater melanin content and thus darker-appearing skin.

### 2.6. Statistical Analysis

To compare the Kairos-derived HRV metrics with ECG-derived HRV metrics, Bland–Altman analyses were conducted. Prerequisites for a traditional Bland–Altman analysis include: (1) the assumption of normality in method difference scores, (2) independence between observed differences and averages (i.e., absence of proportional bias), and (3) constant scatter of difference scores across method averages (scedasticity) [30,31]. The assumption of normality was assessed with normal probability plots, Shapiro–Wilk testing, and skewness indices with a focus on evaluating the presence of skewed distributions [31]. When method difference scores followed a normal distribution, proportional bias was assessed by determining the statistical significance of the slope coefficient in an ordinary least squares (OLS) regression of difference scores against the method averages. In contrast, when difference scores were non-normal, proportional bias was evaluated using the statistical significance of the slope coefficient from a quantile (median) regression of difference scores on method averages. Depending on normality, systematic bias was reported as the mean ± standard deviation or median (interquartile range) when no proportional bias was present, or as an equation using the respective regression coefficients when proportional bias was detected. Scedasticity was assessed by regressing the absolute residuals from the proportional bias assessment model on method averages. Statistically significant slope coefficients were used to indicate heteroscedasticity. When proportional bias was present without heteroscedasticity, the limits of agreement were computed as 1.96 times the standard error of the estimate from the proportional bias regression model [31]. If heteroscedasticity was identified, regardless of proportional bias, V-shaped limits of agreement were calculated by multiplying the regression coefficients from the absolute residuals (from the OLS or median proportional bias model) by 2.46 [30,31]. Relative agreement between methods was quantified with Lin’s concordance correlation coefficient (CCC) [32], where values of <0.90, <0.95, <0.99, and ≥ 0.99 were qualitatively interpreted as poor, moderate, substantial, and near perfect, respectively [33]. Absolute agreement was determined using the mean absolute error (MAE) and mean absolute percent error (MAPE). A MAPE <10% was considered good absolute agreement, while a MAPE >10% was considered poor absolute agreement [34]. Finally, associations were quantified between M-index and method difference scores (Kairos-derived HRV metrics − ECG-derived HRV metrics) with Spearman correlations to assess the impact of skin pigmentation on wristband accuracy. Correlation coefficients were interpreted as trivial (<0.1), small (<0.3), moderate (<0.5), large (<0.7), very large (<0.9), and near perfect (>0.9) [35]. *p* values <0.05 were considered statistically significant. Analyses were conducted using SPSS (Version 27, IBM Corp., Armonk, NY, USA), JMP Pro (Version 16, SAS Institute Inc., Cary, NC, USA), and Excel 2013 (Microsoft Corp., Redmond, WA, USA).

## 3. Results

For supine analyses, five ECG samples were excluded due to ≥ 1 ectopic beats (e.g., premature atrial or ventricular contractions) and six Kairos samples were excluded due to poor signal quality. For seated analyses, six ECG samples were excluded due to ≥ 1 ectopic beats and ten Kairos samples were excluded due to poor signal quality. At least one ECG sample qualified for inclusion (0 ectopic beats) from all but four participants. The final sample sizes were n = 32 and n = 30 for supine and seated comparisons, respectively. Descriptive characteristics of the sample are presented in Table 1.

**Figure 2:**
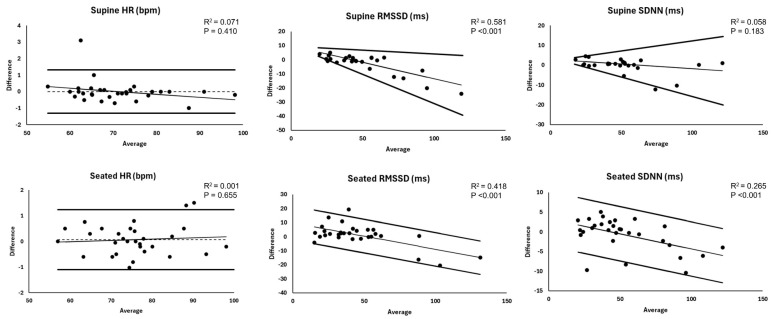
Bland–Altman plots for resting heart rate and time domain heart rate variability metrics. Trend statistics represent simple linear regression results for the assessment of proportional bias. Dashed horizontal line represents mean bias when applicable. RMSSD = root-mean square of successive differences; SDNN = standard deviation of normal RR intervals.

Summary and agreement statistics for the between-device (Kairos versus ECG) comparison of RHR and time domain parameters are reported in Table 2 (supine) and Table 3 (seated), and corresponding Bland–Altman plots are displayed in Figure 2. No significant mean biases were observed for supine and seated RHR, along with near perfect relative agreement, good absolute agreement, no heteroscedasticity or proportional bias, and tight 95% LOA (±<1.5 bpm).

For supine RMSSD, the Kairos wristband exhibited a negative proportional bias and heteroscedasticity, indicating increasing underestimation and greater error at higher RMSSD compared to ECG-derived values. This resulted in V-shaped LOA with a steeper decline for the lower limit line. Relative agreement was substantial, and absolute agreement was good. In the seated position, the Kairos wristband exhibited a negative proportional bias, with increasing underestimation at higher RMSSD values, but no heteroscedasticity. This resulted in parallel but declining LOA. Relative agreement remained substantial, whereas absolute agreement was poor.

For supine SDNN, the Kairos wristband exhibited heteroscedasticity without a proportional bias, resulting in V-shaped LOA. This indicates greater error at higher SDNN compared to ECG-derived values. Relative agreement was substantial, and absolute agreement was good. For seated SDNN, the Kairos wristband exhibited a negative proportional bias, with increasing underestimation at higher SDNN compared to ECG-derived values, but no heteroscedasticity. This resulted in parallel but declining LOA. Relative agreement was near perfect, and absolute agreement was good.

**Figure 3:**
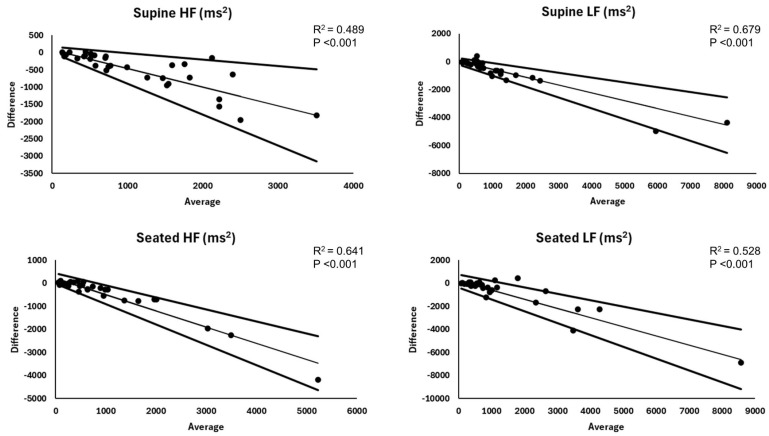
Bland–Altman plots for frequency domain heart rate variability metrics. Trend statistics represent pseudo R squared and method average parameter effect from quantile (median) regression results for the assessment of proportional bias. HF = high frequency; LF = low frequency.

Summary and agreement statistics for the between-device comparison of frequency domain parameters are reported in Table 2 (supine) and Table 3 (seated), and corresponding Bland–Altman plots are displayed in Figure 3. For HF and LF, irrespective of position, the method difference scores deviated from normality and the Kairos wristband exhibited a negative proportional bias and heteroscedasticity, indicating increasing underestimation and greater error at higher HF and LF compared to ECG-derived values. This resulted in V-shaped LOA with a steeper decline for the lower limit line. Relative and absolute agreement were all poor.

**Figure 4:**
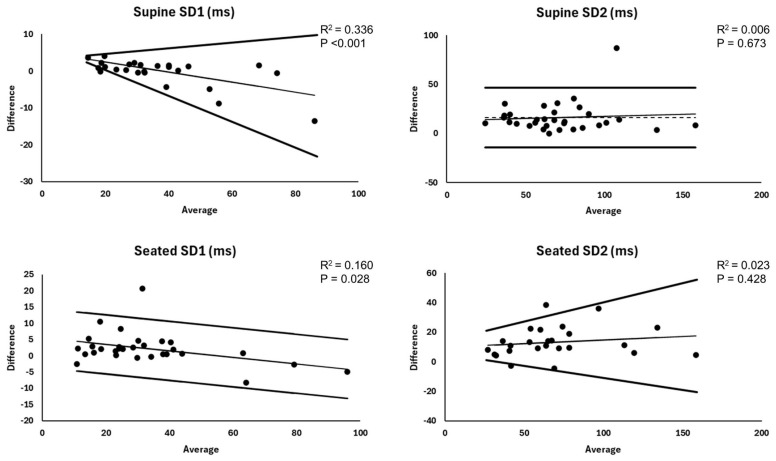
Bland–Altman plots for non-linear heart rate variability metrics. Trend statistics represent simple linear regression results for the assessment of proportional bias. Dashed horizontal line represents mean bias when applicable. SD1 = standard deviation of points perpendicular to the line of identity in the Poincaré plot; SD2 = standard deviation of points along the line of identity in the Poincaré plot.

Summary and agreement statistics for the between-device comparison of non-linear parameters are reported in Table 2 (supine) and Table 3 (seated), and corresponding Bland–Altman plots are displayed in Figure 4. For supine SD1, the Kairos wristband exhibited a negative proportional bias and heteroscedasticity, indicating increasing underestimation and greater error at higher SD1 compared to ECG-derived values. This resulted in V-shaped LOA with a steeper decline for the lower limit line. Relative agreement was substantial, and absolute agreement was good. In the seated position, the Kairos wristband exhibited a negative proportional bias, with increasing underestimation at higher SD1 compared to ECG-derived values, but no heteroscedasticity. This resulted in parallel but declining LOA. Relative agreement remained substantial, whereas absolute agreement was poor.

A significant mean bias was observed for supine SD2 without heteroscedasticity or a proportional bias, indicating that the Kairos wristband systematically underestimated ECG-derived values. In addition, 95% LOA were wide (±30.5 ms), and relative and absolute agreement were poor. In the seated position, the Kairos wristband exhibited heteroscedasticity without a proportional bias, resulting in V-shaped LOA. This indicates greater error at higher SD2 compared to ECG-derived values. Relative and absolute agreement were poor.

**Figure 5:**
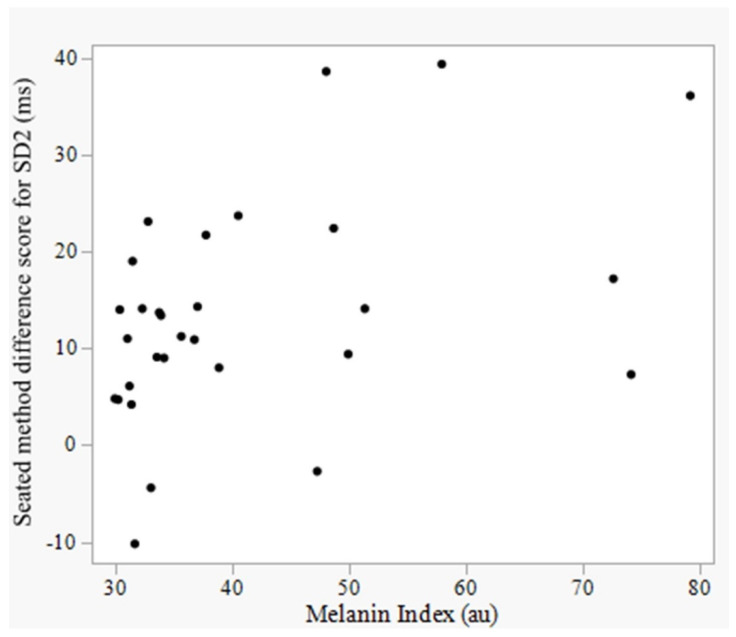
Scatterplot of the association between melanin index and method difference scores (Kairos-derived value – electrocardiography-derived value) for seated SD2. SD2 = standard deviation of points along the line of identity in the Poincaré plot; ECG = electrocardiograph.

A positive association that was moderate in magnitude was observed between seated method difference scores for SD2 (Kairos-derived SD2 – ECG-derived SD2) and M-index (*p* < 0.05, Figure 5). All other associations were non-significant (*p* > 0.05, Table 4). 

## 4. Discussion

This study aimed to (1) evaluate the agreement between the Kairos wristband and gold standard ECG for determining resting HRV parameters, and (2) examine whether increased skin pigmentation was associated with greater estimation errors. Regarding aim 1, excellent agreement was observed for RHR, whereas agreement varied for HRV metrics and recording positions. In general, time domain parameters and SD1 showed superior agreement in comparison to the frequency domain parameters and SD2. However, heteroscedasticity and/or proportional bias was observed for most comparisons. Regarding aim 2, with the exception of one significant association between M-index and seated SD2 difference scores, skin pigmentation generally was not associated with greater error from the Kairos system.

### 4.1. Agreement for Resting Heart Rate

We observed excellent agreement across all statistical methods of comparison between Kairos and ECG for RHR. This finding is consistent with other wristband devices such as the Polar Vantage V2 (supine position) [36], Empatica E4 (seated position) [14], and Apple Watch Series 9 and Ultra 2 (supine position) [37] during standardized 5 min measurements compared to ECG or a Polar H10. In all cases, mean bias values were <1 bpm and 95% LOA were ±~1 bpm. Contrastingly, Fitbit Charge 4 and Samsung Galaxy Watch Active2 demonstrated less precision in estimating supine and seated RHR from 2 min samples (mean bias [95% LOA] range from 0.3 [−16.6 to 16.6] to 4.8 [−33.7 to 43.3] bpm, CCC range from 0.20 to 0.61) [38]. Though more research is needed in larger samples and clinical populations, our findings provide initial support for the use of Kairos in estimating RHR in healthy young adults.

### 4.2. Agreement for Time Domain Heart Rate Variability Parameters

We observed good absolute agreement and substantial, near perfect relative agreement between the Kairos system and ECG for SDNN. However, a small degree of heteroscedasticity was noted in the supine position, indicating increasing Kairos error with higher SDNN, whereas proportional bias was noted in the seated position, reflecting increasing Kairos underestimation at higher SDNN. Thus, the accuracy of Kairos for estimating SDNN diminishes as SDNN values increase. Nevertheless, Kairos showed stronger agreement for SDNN compared with previous investigations. For example, in a comparison of the Garmin Venu 2S GPS smartwatch with ECG during Garmin’s “Health Snapshot” feature (2 min resting measurement), the results showed a higher MAE (9.4 ± 10.1 ms) and MAPE (13.9 ± 13.1%), along with wide 95% LOA (−31.9 below to 20.3 ms above the mean bias of −5.8 ms) [39]. Consistent with the current study, greater underestimation of the criterion was observed at higher SDNN values based on the Bland–Altman plot, though proportional bias was not quantified [39]. Another study showed that the Apple Watch (Series 9 and Ultra 2) showed a significant mean bias of −8.3 ms and wide 95% LOA (−53.8 to 37.2 ms), along with poor absolute agreement (MAE = 20.5 ms, MAPE = 28.9%) during 5 min of supine rest [37]. Moreover, the Bland–Altman figure seems to show heteroscedasticity (greater error with higher SDNN values), though a formal inspection was not conducted [37]. Similarly, the Empatica E4 wristband exhibited a seemingly higher degree of heteroscedasticity based on the Bland–Altman plot when compared with ECG for SDNN during seated 5 min measures [14]. Although the Kairos wristband tended to outperform other consumer-grade wearables, it shares the common limitation of greater error or underestimation at higher SDNN values.

Agreement between Kairos and ECG for RMSSD was weaker than for SDNN. While relative agreement was substantial, absolute agreement was poor, irrespective of position. Moreover, Kairos exhibited proportional bias and heteroscedasticity in the supine position (greater underestimation and error at higher values), and proportional bias in the seated position (greater underestimation at higher values). Relative to the current findings, weaker RMSSD agreement was observed for the Garmin Venu 2S GPS smartwatch during a 2 min “Health Snapshot” (MAE = 12.4 ± 9.6 ms, MAPE = 33.0 ± 37.4%, 95% LOA = −33.3 to 30.4 ms, relative agreement via Pearsons r = 0.89, proportional bias not quantified but visibly present) [39], whereas comparable agreement was observed for the Empatica E4 wristband (significant mean bias of 6.0 ms but narrow 95% LOA of ±8.7 ms, no heteroscedasticity or proportional bias, relative agreement via Pearsons r = 0.92) [14]. A comparison study of the Polar Vantage V2 versus Polar H10 used log transformed RMSSD and reported a significant mean bias (*p* < 0.001), weaker relative agreement (CCC = 0.85), but a smaller MAPE (5.6 ± 6.1%) compared with the current study [36]. Moreover, although proportional bias was directionally consistent with the current study, the Polar Vantage V2 overestimated lower values (trend = −0.70, *p* < 0.001) [36] rather than underestimating higher values, as was the case for Kairos.

### 4.3. Agreement for Frequency Domain Heart Rate Variability Parameters

Very poor agreement was observed between Kairos and ECG for computation of frequency domain parameters (LF and HF) by every statistical method of comparison and in both supine and seated positions. This finding agrees with another investigation in which the Elite HRV smartphone application showed much stronger agreement with ECG for computing time domain versus frequency domain parameters during 10 min of supine rest and paced breathing at 0.1 Hz [40]. Similar findings (less precision in estimating frequency versus time domain) have been reported for the Oura ring [11] and Apple Watch [12], despite the fact that PPG data were manually processed and filtered by the researchers. Though good relative agreement (r values ≥ 0.95) was observed for LF and HF from the Empatica E4 wristband, mean bias values were high (≤91.6 ms^2^), and heteroscedasticity was reported for both parameters [14]. Use of frequency domain HRV is less common in field settings relative to time domain, which may be due to comparatively greater calculation complexity [41], poorer inter-day reliability [42], higher sensitivity to the confounding of respiration rate [43] and reduced signal quality [12], less convenient (i.e., longer) sample duration requirements [1], and less clear physiological interpretation (specifically for LF) [44]. Moreover, the greater computational demands of frequency domain, and thus increased need for more processing power and memory, likely increases battery usage, further deterring their usage by product developers. Collectively, these reasons may explain why many HRV smartphone applications use RMSSD [36,45,46,47] or SDNN [37] as their featured HRV metric. Until stronger agreement for frequency domain metrics is observed, current and previous findings discourage the use of HF and LF in wearable device systems.

### 4.4. Agreement for Non-Linear Heart Rate Variability Parameters

The observed level of agreement between Kairos and ECG for non-linear HRV varied by metric, with greater precision noted for SD1 versus SD2. Notably, supine SD1 showed substantial relative agreement and good absolute agreement, despite exhibiting heteroscedasticity and proportional bias. Contrastingly, poor agreement across all statistical comparisons was observed for SD2, irrespective of position. The reason for this discrepancy is unclear, but may be related to differences in their calculations and respective sensitivity to signal quality. For example, SD1 is derived from short-term variability, reflecting differences in successive inter-beat intervals. Contrastingly, SD2 incorporates both short- and long-term variability by accounting for differences in successive inter-beat intervals and the variance of inter-beat intervals. This added complexity may affect its susceptibility to errors introduced by minor motion artifacts or changes in signal quality. However, this discrepancy is not consistent with other devices. For instance, relative and absolute agreement was higher for SD2 versus SD1 in a comparison between Garmin Vivoactive 4 and ECG in a mixed sample of healthy controls and cardiac patients during 30 min of supine rest [13]. However, PPG signal processing, filtering, and computation of HRV parameters were conducted by the researchers, not automatically by the wearable device system. Nevertheless, our finding of fairly good agreement for SD1, particularly in the supine position, is noteworthy because it is interchangeable with, and therefore could be an alternative to, RMSSD [25].

### 4.5. Effect of Skin Pigmentation on Device Accuracy

In the assessment of method difference scores and skin pigmentation, only one association was statistically significant, reflecting a tendency for Kairos to overestimate seated SD2 as M-index increased (Figure 5). However, the overall lack of consistency in slope directionality, with eight negative and six positive (Table 4), suggests no systematic bias across skin pigmentation values. Moreover, out of 14 correlation tests, approximately 1 would be expected to be statistically significant by chance at a significance level of 0.05. Our findings generally agree with a recent investigation that found no significant systematic effects of skin tone on the accuracy of six different commercial wristbands [48]. However, SD2 and LF were not included in the analysis [48]. Meanwhile, a recent meta-analysis of 10 studies reported mixed and inconclusive findings regarding the effect of darker skin tone on the accuracy of consumer wearable devices [49]. Therefore, further research is needed to explore whether the effect of skin tone or pigmentation on device accuracy varies by HRV metric.

### 4.6. Heteroscedasticity and Proportional Bias

Greater errors with increasing values, reflected in heteroscedasticity or proportional bias, were observed in 5/7 metrics from supine comparisons and 6/7 metrics from seated comparisons. This trend suggests that Kairos is less accurate with increasing HRV. We speculate that this can be explained by the filtering algorithm used by the Kairos system, which may flag large inter-pulse intervals as abnormal. This could lead to overcorrection and a subsequent reduction in HRV magnitude, explaining the observed bias in these metrics. Other factors involved may include differences in sampling frequency and resolution, unidentified differences in the Kairos system’s HRV computation methods, and device compensation for motion artifacts or environmental (e.g., light) interference [50].

### 4.7. Strengths and Limitations

Strengths of the current study include: comparison of device-computed HRV metrics for direct evaluation of the Kairos system’s accuracy in real-world settings; strict inclusion of only normal cardiac cycles to eliminate ectopic beat-driven agreement differences; evaluation of skin pigmentation from a racially diverse sample; assessment in supine and seated positions; inclusion of various commonly used HRV metrics; and inspection for proportional bias and heteroscedasticity. Limitations include the lack of overnight assessment to compare nocturnal HRV, inclusion of only healthy young adults, no consideration for body composition effects, and a relatively small sample of individuals with very high skin pigmentation. These limitations should be addressed in future research.

## 5. Conclusions

Our findings indicate that the Kairos device is suitable for measuring supine or seated RHR in healthy young adults. For HRV assessment, SDNN showed the strongest agreement with the criterion, followed by SD1 and RMSSD. However, agreement weakened as HRV increased for each of these metrics, indicating that individuals with higher HRV may receive less accurate values. This pattern may also imply that increases in HRV, such as those following an intervention, may not be accurately captured by the device, posing challenges for longitudinal tracking. Furthermore, frequency-domain metrics (LF and HF) and the non-linear metric SD2 exhibited poor agreement with the criterion, and should not be used at this time. With the increasing adoption of wearable technology for patient or athlete monitoring and clinical research, our findings highlight an emerging pattern among these devices that directly impacts practitioners. Specifically, error magnitude and directionality tends to be non-uniform across the range of HRV values. Thus, when considering which wearable device to use, we encourage practitioners to seek out validation studies and inspect results carefully, as proportional bias and heteroscedasticity are frequently underreported. Finally, these findings may provide direction for future Kairos software updates which could improve HRV estimation accuracy.

## Figures and Tables

**Table 1:** Descriptive characteristics reported as mean ± standard deviation or median (inter-quartile range).

Descriptor	Supine Comparison (n = 32)	Seated Comparison (n = 30)
Male/Female	13/19	12/18
Age (years)	21.7 ± 3.3	21.4 ± 3.0
Height (cm)	169.1 ± 10.5	169.5 ± 9.5
Mass (kg)	76.3 ± 15.3	75.0 ± 13.3
Melanin Index (au)	35.3 (16.6)	34.9 (16.6)

**Table 2:** Variable mean ± standard deviation or median (inter-quartile range) and method comparison statistics for supine values.

Metric	Device	Mean ± SD or Median (IQR)	β	Bias	LOA (95%)	MAE	MAPE%	CCC
RHR (bpm)	Wristband	71.1 ± 9.5	−0.266	0.0 ± 0.7	−1.3–1.3	0.3 ± 0.6	0.5 ± 0.9	0.99
	ECG	71.1 ± 9.7						
RMSSD (ms)	Wristband	53.3 ± 22.3	−0.762 ***	9.95–0.23 * A	Bias ± 2.46 * (−0.16 + 0.073 * A)	5.1 ± 6.5	8.0 ± 7.2	0.95
	ECG	56.3 ± 28.2						
SDNN (ms)	Wristband	54.0 ± 23.4	−0.241	0.4 ± 4.7	Bias ± 2.46 * (−0.35 + 0.06 * A)	3.0 ± 3.6	5.8 ± 6.4	0.98
	ECG	53.5 ± 24.5						
HF (ms^2^)	Wristband	606.5 (1038.0)	−0.839 ***	81.3–0.540 * A	Bias ± 2.46 * (36.5 + 0.14 * A)	482.8 ± 536.6	30.2 ± 16.5	0.72
	ECG	957.0 (1521.1)						
LF (ms^2^)	Wristband	497.0 (519.3)	−0.954 ***	16.1–0.56 * A	Bias ± 2.46 * (97.4 + 0.09 * A)	703.2 ± 1121.8	42.7 ± 21.9	0.74
	ECG	749.6 (1221.4)						
SD1 (ms)	Wristband	39.7 ± 17.4	−0.580 **	5.17–0.14 * A	Bias ± 2.46 * (−0.86 + 0.87 * A)	2.8 ± 3.4	7.2 ± 7.5	0.97
	ECG	39.9 ± 20.0						
SD2 (ms)	Wristband	79.8 ± 30.8	0.078	16.2 ± 15.6 ***	−14.3–46.7	16.2 ± 15.6	33.5 ± 33.9	0.76
	ECG	63.6 ± 29.6						

RHR = resting heart rate; RMSSD = root-mean square of successive differences; SDNN = standard deviation of normal R-R intervals; HF = high frequency; LF = low frequency; SD1 = standard deviation of points perpendicular to the line of identity in the Poincaré plot; SD2 = standard deviation of points along the line of identity in the Poincaré plot; ECG = electrocardiography; SD = standard deviation; IQR = inter-quartile range; CCC = concordance correlation coefficient; β = beta coefficient; LOA = limits of agreement; A = average value of two methods; Italics typeface = median systematic bias from quartile regression. * indicates *p* < 0.05. ** indicates *p* < 0.001. *** indicates *p* < 0.0001.

**Table 3:** Variable mean ± standard deviation or median (inter-quartile range) and method comparison statistics for seated values.

Metric	Device	Mean ± SD or Median (IQR)	β	Bias	LOA (95%)	MAE	MAPE%	CCC
RHR (bpm)	Wristband	75.9 ± 9.7	0.085	0.1 ± 0.6	−1.3–1.3	0.5 ± 0.4	0.6 ± 0.5	0.99
	ECG	75.8 ± 9.7						
RMSSD (ms)	Wristband	46.3 ± 24.6	−0.646 ***	9.95–0.23 * A	Bias ± 1.96 * 6.10	5.2 ± 5.9	14.4 ± 18.3	0.96
	ECG	45.1 ± 29.7						
SDNN (ms)	Wristband	52.5 ± 26.0	−0.515 *	3.34–0.07 * A	Bias ± 1.96 * 3.52	2.9 ± 2.8	6.1 ± 6.4	0.99
	ECG	53.2 ± 28.1						
HF (ms^2^)	Wristband	494.0 (710.3)	−0.967 ***	208.9–(0.70 * A)	Bias ± 2.46 * (103.6 + 0.07 * A)	483.9 ± 878.5	41.4 ± 47.6	0.70
	ECG	609.1 (1092.6)						
LF (ms^2^)	Wristband	542.5 (1006.5)	−0.926 ***	*209.3–* (0.80 * A)	Bias ± 2.46 * (226.1 + 0.10 * A)	791.8 ± 1461.1	34.1 ± 19.4	0.65
	ECG	698.5 (1084.8)						
SD1 (ms)	Wristband	34.2 ± 19.1	−0.400 *	5.50–0.10 * A	Bias ± 1.96 * 4.64	3.5 ± 4.1	15.9 ± 22.7	0.96
	ECG	32.0 ± 21.0						
SD2 (ms)	Wristband	80.7 ± 36.4	0.150	13.4 ± 11.4 ***	Bias ± 2.46 * (1.84 + 0.09 * A)	14.6 ± 9.8	25.5 ± 17.9	0.89
	ECG	67.3 ± 34.8						

RHR = resting heart rate; RMSSD = root-mean square of successive differences; SDNN = standard deviation of normal R-R intervals; HF = high frequency; LF = low frequency; SD1 = standard deviation of points perpendicular to the line of identity in the Poincaré plot; SD2 = standard deviation of points along the line of identity in the Poincaré plot; ECG = electrocardiography; SD = standard deviation; IQR = inter-quartile range; CCC = concordance correlation coefficient; β = beta coefficient; LOA = limits of agreement; A = average value of two methods; Italics typeface = median systematic bias from quartile regression. * indicates *p* < 0.05. *** indicates *p* < 0.0001.

**Table 4:** Spearman correlation coefficients between melanin index and method difference scores (Kairos-derived value/electrocardiography-derived value) for supine and seated comparisons.

	Supine		Seated	
Parameter	ρ	*p*	ρ	*p*
RHR	−0.034	0.854	−0.132	0.486
RMSSD	−0.195	0.284	−0.067	0.727
SDNN	−0.339	0.058	0.203	0.281
HF	0.057	0.757	−0.214	0.257
LF	0.282	0.118	0.213	0.258
SD1	−0.325	0.070	−0.081	0.670
SD2	0.131	0.474	0.450	0.013 *

RHR = resting heart rate; RMSSD = root-mean square of successive differences; SDNN = standard deviation of normal R-R intervals; HF = high frequency; LF = low frequency; SD1 = standard deviation of points perpendicular to the line of identity in the Poincaré plot; SD2 = standard deviation of points along the line of identity in the Poincaré plot. * indicates *p* < 0.05.

## Data Availability

Data are available upon request from the corresponding author.

## References

[1] Camm A.J., Malik M., Bigger J.T., Breithardt G., Cerutti S., Cohen R.J., Coumel P., Fallen E.L., Kennedy H.L., Kleiger R. (1996). Heart rate variability: Standards of measurement, physiological interpretation and clinical use. Task force of the european society of cardiology and the north american society of pacing and electrophysiology. Circulation.

[2] Tulppo M.P., Kiviniemi A.M., Junttila M.J., Huikuri H.V. (2019). Home monitoring of heart rate as a predictor of imminent cardiovascular events. Front. Physiol..

[3] Flatt A.A., Allen J.R., Keith C.M., Martinez M.W., Esco M.R. (2021). Season-long heart-rate variability tracking reveals autonomic imbalance in american college football players. Int. J. Sports Physiol. Perform..

[4] Plews D.J., Laursen P.B., Kilding A.E., Buchheit M. (2012). Heart rate variability in elite triathletes, is variation in variability the key to effective training? A case comparison. Eur. J. Appl. Physiol..

[5] Flatt A.A., Wilkerson G.B., Allen J.R., Keith C.M., Esco M.R. (2019). Daily heart rate variability before and after concussion in an american college football player. Sports.

[6] Natarajan A., Pantelopoulos A., Emir-Farinas H., Natarajan P. (2020). Heart rate variability with photoplethysmography in 8 million individuals: A cross-sectional study. Lancet Digit. Health.

[7] Grosicki G.J., Culver M.N., McMillan N.K., Cross B.L., Montoye A.H., Riemann B.L., Flatt A.A. (2022). Self-recorded heart rate variability profiles are associated with health and lifestyle markers in young adults. Clin. Auton. Res..

[8] Dhingra L.S., Aminorroaya A., Oikonomou E.K., Nargesi A.A., Wilson F.P., Krumholz H.M., Khera R. (2023). Use of wearable devices in individuals with or at risk for cardiovascular disease in the US, 2019 to 2020. JAMA Netw. Open.

[9] A’Naja M.N., Reed R., Sansone J., Batrakoulis A., McAvoy C., Parrott M.W. (2024). 2024 ACSM worldwide fitness trends: Future directions of the health and fitness industry. ACSM’s Health Fit. J..

[10] Knight S., Lipoth J., Namvari M., Gu C., Hedayati M., Syed-Abdul S., Spiteri R.J. (2023). The accuracy of wearable photoplethysmography sensors for telehealth monitoring: A scoping review. Telemed. e-Health.

[11] Cao R., Azimi I., Sarhaddi F., Niela-Vilén H., Axelin A., Liljeberg P., Rahmani A.M. (2022). Accuracy assessment of oura ring nocturnal heart rate and heart rate variability in comparison with electrocardiography in time and frequency domains: Comprehensive analysis. J. Med. Internet Res..

[12] Hernando D., Roca S., Sancho J., Alesanco Á., Bailón R. (2018). Validation of the apple watch for heart rate variability measurements during relax and mental stress in healthy subjects. Sensors.

[13] Theurl F., Schreinlechner M., Sappler N., Toifl M., Dolejsi T., Hofer F., Massmann C., Steinbring C., Komarek S., Mölgg K. (2023). Smartwatch-derived heart rate variability: A head-to-head comparison with the gold standard in cardiovascular disease. Eur. Heart J. Digit. Health.

[14] Stuyck H., Dalla Costa L., Cleeremans A., Van den Bussche E. (2022). Validity of the empatica E4 wristband to estimate resting-state heart rate variability in a lab-based context. Int. J. Psychophysiol..

[15] Puranen A., Halkola T., Kirkeby O., Vehkaoja A. (2020). Effect of skin tone and activity on the performance of wrist-worn optical beat-to-beat heart rate monitoring. Proceedings of the 2020 IEEE SENSORS.

[16] Al-Halawani R., Qassem M., Kyriacou P.A. (2024). Monte carlo simulation of the effect of melanin concentration on light-tissue interactions in transmittance and reflectance finger photoplethysmography. Sci. Rep..

[17] Christiani M., Grosicki G.J., Flatt A.A. (2021). Cardiac-autonomic and hemodynamic responses to a hypertonic, sugar-sweetened sports beverage in physically active men. Appl. Physiol. Nutr. Metab..

[18] Tarvainen M.P., Ranta-Aho P.O., Karjalainen P.A. (2002). An advanced detrending method with application to HRV analysis. IEEE Trans. Biomed. Eng..

[19] Shaffer F., Ginsberg J.P. (2017). An overview of heart rate variability metrics and norms. Front. Public Health.

[20] Kang J., Chang Y., Kim Y., Shin H., Ryu S. (2022). Ten-second heart rate variability, its changes over time, and the development of hypertension. Hypertension.

[21] Hoshi R.A., Santos I.S., Dantas E.M., Andreão R.V., Schmidt M.I., Duncan B.B., Mill J.G., Lotufo P.A., Bensenor I. (2019). Decreased heart rate variability as a predictor for diabetes—A prospective study of the brazilian longitudinal study of adult health. Diabetes Metab. Res. Rev..

[22] Jandackova V.K., Scholes S., Britton A., Steptoe A. (2024). Midlife heart rate variability and cognitive decline: A large longitudinal cohort study. Int. J. Clin. Health Psychol..

[23] Huang W.-B., Lai H.-Z., Long J., Ma Q., Fu X., You F.-M., Xiao C. (2025). Vagal nerve activity and cancer prognosis: A systematic review and meta-analysis. BMC Cancer.

[24] Huikuri H.V., Stein P.K. (2013). Heart rate variability in risk stratification of cardiac patients. Prog. Cardiovasc. Dis..

[25] Ciccone A.B., Siedlik J.A., Wecht J.M., Deckert J.A., Nguyen N.D., Weir J.P. (2017). Reminder: RMSSD and SD1 are identical heart rate variability metrics. Muscle Nerve.

[26] Hoshi R.A., Pastre C.M., Vanderlei L.C.M., Godoy M.F. (2013). Poincaré plot indexes of heart rate variability: Relationships with other nonlinear variables. Auton. Neurosci..

[27] Flatt A.A., Howells D., Williams S. (2018). Effects of consecutive domestic and international tournaments on heart rate variability in an elite rugby sevens team. J. Sci. Med. Sport.

[28] Esco M.R., Flatt A.A., Nakamura F.Y. (2017). Agreement between a smartphone pulse sensor application and electrocardiography for determining lnRMSSD. J. Strength Cond. Res..

[29] Flatt A.A., Esco M.R. (2016). Heart rate variability stabilization in athletes: Towards more convenient data acquisition. Clin. Physiol. Funct. Imaging.

[30] Ludbrook J. (2010). Confidence in altman–bland plots: A critical review of the method of differences. Clin. Exp. Pharmacol. Physiol..

[31] Bland J.M., Altman D.G. (1999). Measuring agreement in method comparison studies. Stat. Methods Med. Res..

[32] Lawrence I., Lin K. (1989). A concordance correlation coefficient to evaluate reproducibility. Biometrics.

[33] Akoglu H. (2018). User’s guide to correlation coefficients. Turk. J. Emerg. Med..

[34] Singh S., Bennett M.R., Chen C., Shin S., Ghanbari H., Nelson B.W. (2024). Impact of skin pigmentation on pulse oximetry blood oxygenation and wearable pulse rate accuracy: Systematic review and meta-analysis. J. Med. Internet Res..

[35] Hopkins W.G., Marshall S.W., Batterham A.M., Hanin J. (2009). Progressive statistics for studies in sports medicine and exercise science. Med. Sci. Sports Exerc..

[36] Nuuttila O.-P., Korhonen E., Laukkanen J., Kyröläinen H. (2022). Validity of the wrist-worn polar vantage V2 to measure heart rate and heart rate variability at rest. Sensors.

[37] O’Grady B., Lambe R., Baldwin M., Acheson T., Doherty C. (2024). The validity of apple watch series 9 and ultra 2 for serial measurements of heart rate variability and resting heart rate. Sensors.

[38] Nissen M., Slim S., Jäger K., Flaucher M., Huebner H., Danzberger N., Fasching P.A., Beckmann M.W., Gradl S., Eskofier B.M. (2022). Heart rate measurement accuracy of fitbit charge 4 and samsung galaxy watch active2: Device evaluation study. JMIR Form. Res..

[39] Williams K., Jamieson A., Chaturvedi N., Hughes A., Orini M. (2023). Validation of wearable derived heart rate variability and oxygen saturation from the garmin’s health snapshot. Proceedings of the 2023 Computing in Cardiology (CinC).

[40] Vondrasek J.D., Riemann B.L., Grosicki G.J., Flatt A.A. (2023). Validity and efficacy of the elite HRV smartphone application during slow-paced breathing. Sensors.

[41] Buchheit M. (2014). Monitoring training status with HR measures: Do all roads lead to rome?. Front. Physiol..

[42] Gisselman A.S., D’Amico M., Smoliga J.M. (2020). Optimizing intersession reliability of heart rate variability—The effects of artifact correction and breathing type. J. Strength Cond. Res..

[43] Penttilä J., Helminen A., Jartti T., Kuusela T., Huikuri H.V., Tulppo M.P., Coffeng R., Scheinin H. (2001). Time domain, geometrical and frequency domain analysis of cardiac vagal outflow: Effects of various respiratory patterns. Clin. Physiol..

[44] Heathers J.A.J. (2014). Everything hertz: Methodological issues in short-term frequency-domain HRV. Front. Physiol..

[45] Flatt A.A., Esco M.R. (2013). Validity of the ithleteTM smart phone application for determining ultra-short-term heart rate variability. J. Hum. Kinet..

[46] Bellenger C.R., Miller D.J., Halson S.L., Roach G.D., Sargent C. (2021). Wrist-based photoplethysmography assessment of heart rate and heart rate variability: Validation of WHOOP. Sensors.

[47] Stone J.D., Ulman H.K., Tran K., Thompson A.G., Halter M.D., Ramadan J.H., Stephenson M., Finomore V.S., Galster S.M., Rezai A.R. (2021). Assessing the accuracy of popular commercial technologies that measure resting heart rate and heart rate variability. Front. Sports Act. Living.

[48] Bent B., Goldstein B.A., Kibbe W.A., Dunn J.P. (2020). Investigating sources of inaccuracy in wearable optical heart rate sensors. NPJ Digit. Med..

[49] Koerber D., Khan S., Shamsheri T., Kirubarajan A., Mehta S. (2023). Accuracy of heart rate measurement with wrist-worn wearable devices in various skin tones: A systematic review. J. Racial Ethn. Health Disparities.

[50] Mühlen J.M., Stang J., Skovgaard E.L., Judice P.B., Molina-Garcia P., Johnston W., Sardinha L.B., Ortega F.B., Caulfield B., Bloch W. (2021). Recommendations for determining the validity of consumer wearable heart rate devices: Expert statement and checklist of the INTERLIVE network. Br. J. Sports Med..

